# Use of parenteral caffeinum natrio-benzoicum: an underestimated risk factor for HCV transmission in China

**DOI:** 10.1186/s12889-015-2299-8

**Published:** 2015-09-19

**Authors:** Hongqin Xu, Ge Yu, Haibo Sun, Juan Lv, Moli Wang, Fei Kong, Mingyuan Zhang, Xiumei Chi, Xiaomei Wang, Ruihong Wu, Xiuzhu Gao, Jin Zhong, Bing Sun, Jing Jiang, Yu Pan, Junqi Niu

**Affiliations:** Department of Hepatology, The First Hospital of Jilin University, No.71 Xinmin Street, Changchun, 130021 China; Ministry of Education; Key Laboratory of Zoonosis, No.519 Dongminzhu Street, Changchun, 130061 China; Jilin Province Key Laboratory of Infectious Diseases, Laboratory of Molecular Virology, No.519 Dongminzhu Street, Changchun, 130061 China; Institute Pasteur of Shanghai, Shanghai Institutes for Biological Sciences, Key Laboratory of Molecular Virology and Immunology, Chinese Academy of Science, Shanghai, China; Department of Clinical Epidemiology, The First Hospital, Jilin University, No. 71 Xinmin Street, Changchun, 130021 China

**Keywords:** Hepatitis C virus, Prevalence, Parenteral caffeinum natrio-benzoicum abuse, Risk factors

## Abstract

**Background:**

Fuyu city in China has a high prevalence of hepatitis C virus (HCV) infection resulting in a high morbidity and mortality from chronic liver disease and hepatocellular carcinoma. This study was conducted to identify the risk factors for HCV infection in Fuyu city.

**Methods:**

Recruitment of study subjects involved a cross-sectional survey using non-random, convenience sampling. Information on demographic variables, risk factors for HCV infection, clinical manifestations, behavioral practices and family history was collected by administering a questionnaire. Anti-HCV antibody was detected using Abbott ARCHITECT i2000SR. HCV infection was confirmed by HCV-RNA testing by the Roche Taqman HCV test. Univariate and multivariate analyses were performed to identify the factors associated with HCV infection.

**Results:**

Out of 3,228 persons that participated in the survey, 3,219 were enrolled in the study. The prevalence of HCV infection was 42.1 % (1355/3219). Among 734 patients with chronic HCV infection whose HCV-RNA genotyping was performed, genotype 1b was the most common (58.0 %), followed by genotype 2a (40.2 %), while co-infection with genotypes 1b and 2a was detected in 1.8 % of the subjects. On univariate analysis, male gender, older age, parenteral caffeinum natrio-benzoicum and share syringes (PCNBSS), and nine other factors were significantly associated with HCV infection. After adjusting for potential confounders, male gender, old age, cigarette smoking, lower education level, history of blood transfusion, blood donation, prior dental surgery, and PCNBSS were found to be independently associated with HCV infection.

**Conclusions:**

The prevalence of HCV infection is likely to be high among residents in Fuyu and we observed that genotypes 1b and 2a dominated in the city. Our findings support the hypothesis that PCNBSS which became endemic in Fuyu city during 1970s-1980s is strongly associated with HCV positivity.

**Electronic supplementary material:**

The online version of this article (doi:10.1186/s12889-015-2299-8) contains supplementary material, which is available to authorized users.

## Introduction

In recent years, a majority of patients with hepatitis C virus (HCV) infection admitted at our hospital were residents of Fuyu City, which is located in Northwest region of Jilin Province, China. The city has a heavy burden of liver disease, most likely attributable to chronic HCV infection [1]. According to official figures, the incidence of HCV infection in Jilin province in year 2012 was higher (0.31 ‰) than the average incidence reported in the mainland China (0.16 ‰) [2]. A rampant use of parenteral caffeinum natrio-benzoicum with shared syringes (PCNBSS) during mass celebrations is the likely mode of transmission of blood-borne pathogens, including HCV in Fuyu city.

Caffeinum natrio-benzoicum (CNB), also known as caffeine and sodium benzoate, is used as a psychotropic drug. Caffeine has an excitatory effect on the nervous system, while sodium benzoate assists in solubilization and absorption of caffeine. In the 1970s, certain population groups in north, northeast and northwest regions of China started abusing CNB through oral or nasal inhalation (snorting). Around the same time, residents in Fuyu developed the habit of recreational use of parenteral CNB during community celebrations. Similar recreational use of CNB has not been reported from any other part of the world. Parenteral abuse of CNB during celebrations has earlier been implicated as a risk factor for acquisition of HCV and hepatitis B virus (HBV) infection in China [3]. Caffeinum natrio-benzoicum has been listed as a psychotropic drug and its abuse has been forbidden in China since 1988.

We investigated the relative importance of PCNBSS in the spread of HCV infection in the Fuyu City by comparing cumulative lifetime exposure to PCNBSS with the current specific prevalence of HCV infection. We hypothesized that the parenteral abuse of CNB during celebrations coupled with inadequately sterilized drug injecting paraphernalia was a major factor in the spread of HCV infection in Fuyu City.

## Methods

### Study population and recruitment

The target population was permanent residents in Fuyu City. In September 2012, we conducted a cross-sectional survey using non-random, convenience sampling in Fuyu City, Jilin Province, China. In the first stage, five survey locations (Township Health Center) were obtained from all the 5 towns in Fuyu City (Desheng, Gengxin, Wanfa, Dalinzi and Gongpengzi). In the second stage, more than 100 village committee members and rural doctors in all the 5 towns in Fuyu City were recruited and trained for two days on effective publicity for the survey. They then publicized the study in their village using recruitment cards and flyers which was necessary for the investigators to perform cluster sampling. In the third stage, our research team performed the survey for 5–8 days at each survey location. Enrolled participants were encouraged to inform their peers about the study.

Individuals were eligible to participate, if they were current residents of Fuyu city, and consented to undergo laboratory investigations for HCV, HBV and HIV. After screening for eligibility and obtaining informed consent, each subject was made to complete a questionnaire. Participants who did not complete the questionnaire were excluded from the study. Subjects were asked to return 4–6 weeks later to receive the laboratory test results. Those subjects who were diagnosed with HCV and/or HBV infection were referred to a medical center for further treatment and care.

### Data collection

Each subject was asked to complete a questionnaire that included information on demographic variables (age, gender and race), risk factors for HCV infection, clinical manifestations, behavioral activity, and family history. Clinical information included the presence or absence of diabetes, thyroid disease, autoimmune disorders, previous history of surgery, blood transfusion, blood donation, and antiviral treatment, and family history of liver disease. Behavioral information regarding injection drug use (especially parenteral CNB, frequency of use, and date of first/last injection), alcohol abuse, and cigarette smoking was collected. The subject’s age at the time of first use of injectable drug was also recorded.

### Virological detection

Blood samples were tested for Hepatitis B surface antigen (HBsAg), and antibodies against HBsAg (anti-HBsAg), Hepatitis B core antigen (anti-HBc),Hepatitis C (Anti-HCV) and HIV using Abbott ARCHITECT i2000SR Immunoassay System (Abbott Laboratories; Abbott Park, IL, USA) at the clinical laboratory of the First Hospital of Jilin University. All samples that were anti-HCV positive were confirmed by HCV-RNA test (COBAS AmpliPrep/TaqMan, Roche Diagnostics Ltd, Rotkreuz, Switzerland). Anti-HCV positive results were confirmed by recombinant immune blot assay (CHIRON RIBA HCV 3.0 SIA, Ortho Clinical Diagnostics, Johnson & Johnson, USA) in individuals who subsequently tested negative for HCV-RNA. HCV genotyping was performed by multicolor fluorescence polymerase chain reaction (PCR) using an HCV-RNA genotyping kit (BioAssay Science & Technology Co. Ltd., Beijing, China).

### Statistical analysis

Statistical analyses were performed using the statistical software R for Windows, version 3.1.1. Continuous variables were expressed as median and inter-quartile range (IQR) or mean and standard deviation, as appropriate, and were compared using the Student *t*-test or the Mann–Whitney U-test. Categorical variables were compared using the Chi-square test or the Fisher’s exact test. Variables with statistical significance (*P* ≤0.05) in the univariate model were analyzed using a multivariate logistic regression model. All variables found to be significant (*P* ≤0.05) through univariate analysis were considered for inclusion in multivariable analysis. A backward stepwise logistic regression was performed, and factors that were statistically significant (*P* ≤0.05) in multivariable analysis remained in the final model. A *P*-value ≤0.05 was considered statistically significant; Odds ratio (OR) with 95 % Confidence Interval (CI) are presented to demonstrate the strength and direction of these associations.

### Ethical considerations

The study was approved by the Ethics Committee at the First Hospital of Jilin University, Changchun, China. Written informed consent was obtained from all participants before initiating any study-specific procedure. Due care was taken to incorporate specific protocols aimed at maintaining data confidentiality, as well as to protect the subjects against potential ethical violation. Data collection was usually conducted at the township Health Center. At the time of filling of questionnaire by the participants, presence of both interviewee and interviewer was made mandatory. All necessary precautions for obtaining data confidentially were taken (the questionnaire was applied only in the presence of the interviewee and interviewer). Those agreeing to take part in the study were asked to have their blood collected at the university hospital laboratory for further evaluation of anti-HCV and anti-HBV activity, always in the presence of the researcher.

## Results

### Demographic characteristics and prevalence of HBV, HCV and HIV infection

Out of 3,228 persons who participated in the survey, 3,219 (male, 1536 [47.7 %]; female, 1683 [52.3 %]) were enrolled in the study. Data pertaining to 9 participants was excluded due to incomplete or unreliable information. Out of 3219 study subjects, 2,182 (67.8 %) were aged between 40 and 60 years. Overall, the prevalence of HCV and HBV mono-infection was 39.8 % (95 % CI: 38.1 %–41.5 %) and 5 % (95 % CI: 4.2 %–5.8 %) respectively, the prevalence of HBV/HCV co-infection was 2.9 % (95 % CI: 2.3 %–3.5 %). HCV-RNA genotyping was performed on 873 subjects with chronic HCV infection. There were 139 subjects with chronic HCV infection in whom HCV-RNA genotyping was not possible due to low HCV-RNA levels. Genotype 1b was the most common HCV genotype (426/734, 58.0 %) followed by genotype 2a (295/734, 40.2 %). Co-infection with HCV genotypes 1b and 2a was detected in 1.8 % subjects. Hepatitis C virus load range from 0 IU/ml to 111700000 IU/ml and the median viral load was 577000 IU/ml among HCV positive participants (Clinical and virological characteristics of the HCV positive participants are supplied in Additional file 1). None of the study subjects tested positive for HIV infection.

### Univariate analysis of variables associated with HCV infection

The results of univariate analysis are presented in Table 1. The results showed that 795 (24.7 %) participants were exposed to PCNBSS in their lifetime. Participants with a history of exposure to PCNBSS were more likely to get HCV infection (73.5 %). Male gender (OR = 2.17, 95 % CI: 1.89–2.51), older age (OR = 3.27, 95 % CI: 2.83–3.79), lower educational level (OR = 4.21, 95 % CI: 1.48–5.72; OR = 5.92, 95 % CI: 3.19–10.99), cigarette smoking (OR = 2.17, 95 % CI: 2.35–3.13), alcohol consumption (OR = 2.09, 95 % CI: 1.80–2.42), history of blood transfusion (OR = 1.71, 95 % CI: 1.17–2.50), prior dental surgery (OR = 1.49, 95 % CI: 1.29–1.71), and PCNBSS (OR = 5.93, 95 % CI: 4.96–7.10) were significantly associated with an increased risk of HCV infection. Factors negatively associated with HCV infection were non-farming occupation (OR = 0.23, 95 % CI: 0.16–0.33), ear piercing (OR–0.54, 95 % CI: 0.47–0.62), blood donation (OR–0.35, 95 % CI: 0.20–0.59), and tattoo (OR = 0.34, 95 % CI: 0.22–0.53).

**Table 1:** Univariate analysis of socio-demographic characteristics by HCV status among residents in Fuyu City, China

Variable		Total N (%)	HCV + N (%)	Odds ratio (95 % CI)	*χ* ^*2*^	*P* value
Sex	female	1683 (100)	558 (33.2)	2.17 (1.89–2.51)	115.6	<0.001
	Male	1536 (100)	797 (51.9)			
Age	<50 years old	1575 (100)	438 (27.8)	3.27 (2.83–3.79)	258.2	<0.001
	>50 years old	1644 (100)	917 (55.8)			
Education	college	138 (100)	11 (8.0)		55.4	<0.001
	middle school	805 (100)	270 (33.5)	4.21 (2.24–7.90)		
	primary school	2276 (100)	1074 (47.2)	5.92 (3.19–10.99)		
Occupation	farmer	3032 (100)	1327 (43.8)	0.23 (0.16–0.33)	59.9	<0.001
	non-farmer	187 (100)	28 (15.0)			
Cigarette smoking	No	1684 (100)	517 (30.7)	2.17 (2.35–3.13)	188.1	<0.001
	Yes	1535 (100)	838 (54.5)			
Alcohol consumption	No	2138 (100)	771 (36.1)	2.09 (1.80–2.42)	95.5	<0.001
	Yes	1081 (100)	584 (54.1)			
HBV infection	no	2966 (100)	1262 (42.5)	0.785 (0.602–1.024)	2410	<0.001
	yes	253 (100)	963 (36.7)			
PCNBSS	no	2424 (100)	771 (31.8)	5.93 (4.96–7.10)	426.1	<0.001
	yes	795 (100)	584 (73.5)			
Surgery	no	1814 (100)	771 (42.5)	0.96 (0.84–1.11)	0.285	0.59
	yes	1405 (100)	584 (41.6)			
Ear piercing	no	1927 (100)	925 (48)	0.54 (0.47–0.62)	71.1	<0.001
	yes	1292 (100)	429 (33.2)			
Blood transfusion	no	3108 (100)	1293 (41.6)	1.71 (1.17–2.50)	9.2	<0.05
	yes	111 (100)	61 (55)			
Blood donation	no	3136 (100)	1338 (42.7)	0.35 (0.20–0.59)	16.3	<0.001
	yes	83 (100)	17 (20.5)			
Tattooing	no	3101 (100)	1331 (42.9)	0.34 (0.22–0.53)	24.5	<0.001
	yes	118 (100)	24 (20.3)			
Prior dental surgery	no	1666 (100)	623 (37.4)	1.489 (1.29–1.71)	33.6	<0.001
	yes	1551 (100)	730 (47.1)			

HCV, Hepatitis C virus; PCNBSS, Parenteral caffeinum natrio-benzoicum and share syringes; CI, Confidence Interval

### Multivariate analysis of variables associated with HCV infection

Gender, age, cigarette smoking, PCNBSS, occupation, education, blood transfusion, blood donation, prior dental surgery, ear piercing, tattooing, alcohol consumption, and HBV infection, which were associated with HCV infection in univariate analysis, were considered for entry in multivariable analysis. After adjusting for potential confounders, male gender (OR = 2.06, *P* <0.001), older age (OR = 2.78, *P* <0.001), cigarette smoking (OR = 1.85, *P* <0.001), lower educational level (RR = 2.13, *P* <0.001), history of blood transfusion (OR = 1.68, *P* <0.05), prior dental surgery (OR = 1.21, *P* <0.05), and PCNBSS (OR = 4.90, *P* <0.001) were independently associated with HCV infection on multivariate analysis (Table 2). The most significant risk factor found in our study was PCNBSS, with a prevalence of 24.7 % among study subjects. Ear piercing, tattoo, alcohol consumption, occupation and HBV infection were not found to be statistically associated with HCV infection in this study.

**Table 2:** Multivariate regression analysis of factors associated with Hepatitis C virus infection among residents in Fuyu City, China

Variable	B	S.E.	Wald	*P* value	OR	95 % CI for OR
Sex	0.72	0.09	66.77	<0.001	2.06	1.73–2.45
Age	1.02	0.09	141.76	<0.001	2.78	2.35–3.95
Cigarette smoking	0.61	0.08	52.82	<0.001	1.85	1.57–2.18
PCNBSS	1.59	0.10	252.87	<0.001	4.90	4.03–5.96
Prior dental surgey	0.19	0.08	5.20	<0.05	1.21	1.03–1.43
Blood transfusion	0.52	0.22	5.45	<0.05	1.68	1.09–2.61
Education						
Primary school	Reference	Reference	Reference	Reference	Reference	Reference
Middle school	−0.70	0.10	45.37	<0.001	0.50	0.41–0.61
College	−1.83	0.34	29.43	<0.001	0.16	0.08–0.31
Blood donation	−0.76	0.34	4.95	<0.05	0.47	0.24–0.91
Constant	−0.47	0.42	1.26	0.26	0.62	

PCNBSS, parenteral caffeinum natrio-benzoicum and share syringes; S.E., Standard Error

## Discussion

In this study, the prevalence of HCV infection in Fuyu City was found to be 42.7 %, which was approximately 100-fold higher than the national rate (0.43 %) [4, 5]. The most common genotypes of HCV circulating in Fuyu City were 1b and 2a, which is consistent with the other studies conducted in East Asia [6, 7]. Several studies have reported a higher risk of HCV infection in professional blood donors, patients on hemodialysis, hemophiliacs, injection drug users (IDUs), men who have sex with men, and those with multiple sex partners [8–11]. In the present study, important risk factors associated with HCV transmission were PCNBSS, history of blood transfusion and prior dental surgery. The other significant risk factors for transmission of HCV included farming as an occupation, older age, male gender, and cigarette smoking. The risk factor most strongly associated with HCV infection was PCNBSS which supports the hypothesis that PCNBSS was an important predisposing factor for establishment of a large reservoir of HCV infection in Fuyu City. The results of the regression analysis confirmed the strong association between PCNBSS and HCV infection, even after adjusting for other confounding variables.

In our earlier study conducted in Changchun ling, a village near Fuyu City, where the custom of PCNBSS abuse during ceremonies was not practiced, the prevalence of HCV infection was found to be only 3.9 % [1]. These contrasting findings strongly implicate PCNBSS as a factor responsible for the wide variability in the prevalence of HCV infection in two geographically contiguous areas.

In this study, prevalence of HCV infection was higher in males >50 years old. This is consistent with the assumption that recreational use of PCNBSS started in 1970s and 1980s when these people were old enough to participate in the wedding and funeral ceremonies, during which exposure to PCNBSS occurred. According to the national epidemiological survey of viral hepatitis conducted in China from 1991 to 1995, there was no significant difference in the prevalence of HCV infection between males and females [12]. Similar findings of a higher HCV prevalence in elderly males has also been reported from an Egyptian study [13]. However, a Taiwanese study involving 23820 participants reported a higher sero-prevalence of HCV infection in females than in males [14]. In our study, HCV infection rate in males (51.9 %) was higher than that in females (33.2 %), while the males had an increased chance of exposure to PCNBSS.

In the multivariate model, a history of blood transfusion and prior dental surgery were significant risk factors associated with HCV infection, which suggests that unsafe medical practices may have contributed to HCV transmission. Since limited resources are available for prevention of HCV infection, the focus should be directed on ensuring implementation of safe practices in healthcare settings. To prevent nosocomial infection, mandatory screening of blood donors for HCV should be enforced. The Chinese government has prohibited the use of paid blood donors since 1998, which has improved blood safety [15]. This transition in the blood donor recruitment method has been associated with a gradual decline in the prevalence of anti-HCV among blood donors, which is also reflected in the low prevalence of HCV infection in the blood donors participating in our study.

In addition to syringe-sharing, sharing of injecting paraphernalia is a well-recognized risk factor for HCV infection [16]. However, in the present study ear piercing and tattooing were significantly associated with a decreased risk of HCV infection on univariate analysis. Since the older ages and male gender are risk factors for HCV infection in our study, this outcome could have resulted from the influence of age and sex (perhaps females and youngsters were more likely to be involved in these practices). After adjusting for ages, gender and other potential confounders, the association between a history of ear piercing or tattooing was not significant on multivariate analysis. As PCNBSS is reported popular in older (>50 years) male group, all of the above can deem that PCNBSS was the independent risk factor of HCV transmission in Fuyu city.

Since prevention messages against PCNBSS are more prevalent than messages advising against paraphernalia sharing, the residents in Fuyu may lack the awareness of prevention HCV from these factors such as ear piercing or tattooing.

Several studies have demonstrated a significant association between lower literacy level and HCV infection [17–19]. In the present study, people educated up to middle or primary school education had a higher prevalence of HCV infection as compared to those who had completed college education. Further, HCV infected individuals were more likely to have unhealthy habits like cigarette smoking, which is also consistent with other studies [20].

Currently available antiviral regimes for treatment of HCV infection have a high efficacy with a sustained virological response rate close to 100 % if treatment is started early during the acute phase [21]. Unfortunately, most cases of HCV are diagnosed when chronic infection is already established, which makes spontaneous viral clearance difficult in these patients. Some HCV positive subjects were not aware of their seropositive status before their enrollment in this study. It is likely that in these subjects, the risk behaviors for HCV transmission may have been influenced by their new found seropositive status, possibly contributing to minimizing other risky behavior such as sharing toothbrush with families. Furthermore, these HCV positive subjects were willing to get earlier treatment to prevent the development of chronic liver disease and hepatocellular carcinoma. Therefore, there is a pressing need for one-time screening for HCV infection in populations with high-risk behavior.

PCNBSS appears to be the most important risk factor for HCV infection in Fuyu. Other factors also contribute to the transmission of HCV in the city. Ongoing research is required to assess the emerging risk of transmission. Most of the HCV infected subjects in this study were males. Further research is needed to understand how familial transmission occurs with regard to spouses and children. Given the high risk of infection transmission among family members of HCV-infected individuals, awareness raising activities are urgently needed.

Use of a non-random convenience sample and self-reporting of at-risk behaviors by subjects are key limitations of our study. The study sample may not be representative of the general population in Fuyu. Similarly self-reporting by subjects could have been affected by recall bias. In order to minimize recall bias, questions related to substance use and injection behavior recall (such as when and where did you take the PCNBSS) were kept in the questionnaire. Despite these limitations, the survey demonstrated a high prevalence of HCV infections in Fuyu city where PCNBSS was most important risk factors for HCV transmission.

## Conclusion

The prevalence of HCV infection is likely to be high among residents in Fuyu and we observed that genotypes 1b and 2a dominated in the city. Our findings support the hypothesis that PCNBSS which became endemic in Fuyu city during 1970s–1980s is strongly associated with HCV positivity. More attention should be paid to routine screening for HCV in high-risk areas.

## Additional file

Additional file 1:
**Clinical and virological characteristics of the HCV positive participants.** (DOCX 18 kb)

## Abbreviations

HCV: Hepatitis C virus
PCNBSS: Parenteral caffeinumnatrio-benzoicum and shared syringes
HBV: Hepatitis B virus
HBsAg: Hepatitis B surface antigen
anti-HBsAg: Antibody to Hepatitis B surface antigen
anti-HBc: Antibody to hepatitis B core antigen
RNA: Ribonucleic acid
ELISA: Enzyme-linked immune sorbent assay
OR: Odds ratio
CI: Confidence interval
IQR: Inter-quartile range
S.E.: Standard error
