# Supplementary material for: Use of parenteral caffeinum natrio-benzoicum: an underestimated risk factor for HCV transmission in China
Source: BMC Public Health. 2015 Sep 19;15:928. doi: 10.1186/s12889-015-2299-8 (PMC4575778; doi:10.1186/s12889-015-2299-8)
Supplement: Additional file 1: — Clinical and virological characteristics of the HCV positive participants. (DOCX 18 kb) [file 12889_2015_2299_MOESM1_ESM.docx]

| Characteristics | | Median (P25,P75) | N (%) | reference ranges |
| --- | --- | --- | --- | --- |
| Age |  | 54.5(48.5, 60.2) |  |  |
| Gender |  |  |  |  |
|  | Male |  | 797(58.8%) |  |
|  | Female |  | 558(41.2%) |  |
| HCV Genotype * | |  |  |  |
|  | 1a |  | 426(58.0%) |  |
|  | 2b |  | 295(40.2%) |  |
|  | 1a/2b |  | 13(1.8%) |  |
| HCV load (log_10_ IU/ml) | | 5.76(2.44,6.77) |  |  |
| Liver function |  |  |  |  |
|  | AST (U/L) | 35(25,58) |  | 8-40 |
|  | ALT (U/L) | 37(22,67.8) |  | 8-50 |
|  | ALP(U/L) | 77(64,94.8) |  | 15-112 |
|  | GGT(U/L) | 39(22,80) |  | 5-54 |
|  | Total Protein(g/l) | 77(73.3,81) |  | 60-83 |
|  | Albumin (g/L) | 44.9(42.6,47.4) |  | 35-55 |
|  | total bilirubin(μmol/L) | 13.6(9.8,18.5) |  | 6.80-30.00 |
|  | Direct Bilirubin(μmol/L) | 4.3(3.2,5.7) |  | 0-8.60 |
|  | Cholinesterase (U/L) | 7999(6488,9620) |  | 4300-12000.00 |
|  | Platelet (10^9^/L) | 185(145,226) |  | 100-300 |

**Additional Table.1** Clinical and virological characteristics of the HCV positive participants

*HCV-RNA genotyping was performed on 873 subjects with 139 was typeable due to low HCV-RNA levels

ALT, alanine aminotransferase; AST, aspartate aminotransferase; GGT, glutamyl transpeptidase;

ALP, alkaline phosphatase
